# A *Drosophila* model linking diet-induced metabolic disease and cancer

**DOI:** 10.1186/2049-3002-2-S1-O19

**Published:** 2014-05-28

**Authors:** Susumu Hirabayashi, Thomas Baranski, Ross Cagan

**Affiliations:** 1 Department of Developmental and Regenerative Biology, Icahn School of Medicine at Mount Sinai, New York, NY 10029, USA; 2 Department of Medicine, Washington University School of Medicine, St. Louis, MO 63110, USA

## Background

Epidemiological studies have provided strong evidence for the association between cancer and metabolic diseases including obesity and diabetes, but the underlying mechanism remains poorly understood.

## Methods

Feeding *Drosophila* a diet high in sucrose was previously demonstrated to direct important aspects of type 2 diabetes including insulin-resistance, hyperglycemia, increased insulin levels, accumulation of fat, and heart dysfunction [1,2]. We used this model to explore the effects of high dietary sucrose on tumor progression of Ras/Src co-activated *Drosophila* tumor model.

## Results

We demonstrate that high dietary sucrose, but not high dietary fat transforms Ras/Src-activated cells from localized growths to aggressive tumors with emergent metastases. Surprisingly, while most tissues displayed aspects of metabolic dysfunction including insulin resistance, Ras/Src-activated tumors retained insulin pathway sensitivity and exhibited an increased ability to import glucose. We provide evidence that this reflects increased insulin signaling, which in turn acts through Wingless/Wnt signaling to promote diet-mediated malignant phenotypes within Ras/Src-activated tumors [3]. These fly models should provide useful paradigms to study the link between metabolic dysfunction and tumorigenesis in the context of a whole animal.
